# Endosomal dysfunction in iPSC-derived neural cells from Parkinson’s disease patients with VPS35 D620N

**DOI:** 10.1186/s13041-020-00675-5

**Published:** 2020-10-08

**Authors:** Keiko Bono, Chikako Hara-Miyauchi, Shunsuke Sumi, Hisayoshi Oka, Yasuyuki Iguchi, Hirotaka James Okano

**Affiliations:** 1 Division of Regenerative Medicine, The Jikei University School of Medicine, 3-25-8 Nishi-Shimbashi, Minato-ku, Tokyo, 105-8461 Japan; 2 Department of Neurology, The Jikei University School of Medicine, 3-25-8 Nishi-Shimbashi, Minato-ku, Tokyo, 105-8461 Japan; 3 Department of Neurology, Daisan Hospital, The Jikei University School of Medicine, 4-11-1 Izumihoncho, Komae-shi, Tokyo, 201-8601 Japan

**Keywords:** Parkinson’s disease, iPSC, VPS35, Retromer, Endosomes

## Abstract

Mutations in the Vacuolar protein sorting 35 (*VPS35*) gene have been linked to familial Parkinson’s disease (PD), PARK17. VPS35 is a key component of the retromer complex, which plays a central role in endosomal trafficking. However, whether and how VPS35 deficiency or mutation contributes to PD pathogenesis remain unclear. Here, we analyzed human induced pluripotent stem cell (iPSC)-derived neurons from PD patients with the VPS35 D620N mutation and addressed relevant disease mechanisms. In the disease group, dopaminergic (DA) neurons underwent extensive apoptotic cell death. The movement of Rab5a- or Rab7a-positive endosomes was slower, and the endosome fission and fusion frequencies were lower in the PD group than in the healthy control group. Interestingly, vesicles positive for cation-independent mannose 6-phosphate receptor transported by retromers were abnormally localized in glial cells derived from patient iPSCs. Furthermore, we found α-synuclein accumulation in TH positive DA neurons. Our results demonstrate the induction of cell death, endosomal dysfunction and α -synuclein accumulation in neural cells of the PD group. PARK17 patient-derived iPSCs provide an excellent experimental tool for understanding the pathophysiology underlying PD.

## Introduction

Parkinson’s disease (PD), the second most common neurodegenerative disorder after Alzheimer’s disease (AD), affects more than 2% of adults over 60 [1, 2]. PD is characterized by progressive motor symptoms such as bradykinesia, resting tremor, muscular rigidity, and postural instability (these four symptoms are called “parkinsonism”), as well as nonmotor symptoms such as olfactory dysfunction, autonomic dysfunction, and dementia [3]. The pathological hallmark of PD is the loss of dopaminergic (DA) neurons in the substantia nigra pars compacta and the presence of Lewy bodies, which consist of aggregated α-synuclein protein [4]. Lewy bodies are also found not only in DA neurons in the substantia nigra but also more broadly in the PD brain [5].

PD is predominantly an idiopathic disease, with the largest risk factor being simply age; however, up to 10% of cases occur in a familial manner by both autosomal dominant and recessive transmission. Mutations in several pathogenic genes have been identified over the last two decades and found to be associated with both familial and sporadic PD. For example, mutations in *α-synuclein* (also called PARK1) and leucine-rich repeat kinase 2 (*LRRK2* or PARK8) cause autosomal dominant PD, and mutations in *parkin* (PARK2), *DJ-1* (PARK7), *Pink1* (PARK6), and *ATP13A2* (PARK9) have been linked to autosomal recessive PD [6–8]. Investigation into the functions of these genes and mutant proteins has revealed the pathophysiological mechanisms of both familial and sporadic PD [6, 8, 9].

Vacuolar protein sorting 35 (*VPS35*, also called PARK17) was reported to be a pathogenic gene for late-onset autosomal dominant PD. A single missense mutation, c.1858G > A (p.D620N), was originally shown to segregate with PD in Swiss and Austrian families and has been identified in several PD subjects and families worldwide [10, 11]. *VPS35* mutation is the second most common cause of late-onset familial PD after *LRRK2* mutations. Additional rare *VPS35* variants (i.e., p.M57I, p.I241M, p.P316S, p.R524W, p.A737V, and p.L774M) may also be linked to PD, although their pathogenicity remains unclear. The mean age of onset of PD in patients with the VPS35 mutation is 53 years [11], and the clinical symptoms of these patients closely resembled those of the idiopathic form of PD, which manifests as tremor-dominant dopa-responsive parkinsonism [12]. One autopsy case of PARK17 was reported in Japan. There were no Lewy bodies in DA neurons in the substantia nigra pars compacta, but phosphorylated α-synuclein had aggregated in the neurons of the substantia nigra, locus coeruleus, dorsal vagal nucleus, nucleus basalis of Meynert, and cardiac muscle. This distribution of α-synuclein is similar to that observed in sporadic PD [13].

VPS35 is a key component of the retromer complex [14–18]; it contains two protein subcomplexes: a cargo-selective subcomplex that consists of a trimer of VPS35, VPS29, and VPS26 and a membrane deformation subcomplex that consists of sorting nexin dimers [15, 16]. The most widely characterized role of the retromer is recycling of transport proteins back to the trans-Golgi network (TGN) in the endosomal trafficking system [19–21]. Numerous transmembrane proteins/receptors, including cation-independent mannose 6-phosphate receptor (CI-MPR) [21], amyloid precursor protein (APP) [22], APP processing β1 secretase [23], Wntless [24–26], β2-adrenergic receptor [27], and AMPA-type glutamate receptors [28], have been identified as retromer cargos. New evidence indicates that the retromer is a “master conductor” of endosomal sorting and trafficking [14]. The retromer complex plays a central role in endosomal trafficking, and retromer dysfunction has been linked to neurological disorders, such as PD and AD [29].

Endosomal trafficking is essential for the maintenance of cellular homeostasis and plays a crucial role in the trafficking of proteins through the cellular endomembrane system. Neurons are heavily dependent on such protein trafficking processes by endosomes. Following its internalization at the plasma membrane by endocytosis, the cargo is delivered to the early endosome, where sorting occurs. This trafficking step is highly selective and involves a series of membrane fusion/fission events mediated by specific GTPases. The maturation from early to late endosome occurs as a continuum associated with an increase in the number of intraluminal vesicles (multivesicular bodies; MVBs), luminal acidification, and endosome movement from the cell periphery toward the nucleus [30–32]. This morphological maturation is associated with a molecular switch in GTPase composition with the loss of Rab5 expression and acquisition of Rab7 [33]. The small GTPase Rab5 is a marker for the early endosome and a key regulator of endosomal trafficking processes. The small GTPase Rab7 is known to be a marker of late endosomes. It has now been shown that Rab7a is required for recruitment of the cargo-selective retromer complex [17, 34, 35].

Genetic discoveries have started to illuminate cellular pathways and functions that are involved in the development of PD, and impaired intracellular trafficking is emerging as a mechanistic link between many PD-associated genes in the endosomal trafficking machinery and lysosomes [36]. A number of PD-associated genetic mutations and polymorphisms disrupt protein trafficking and degradation through the endosomal pathway, and how such defects could arise from or contribute to the accumulation and misfolding of α-synuclein in Lewy bodies has been discussed [37].

In the present study, we generated patient-specific iPSC-derived DA neurons from PD patients with the VPS35 D620N mutation and healthy individuals. To understand the role of the retromer in the endosomal trafficking system, we observed the intracellular behavior of endosomal vesicles by live-cell fluorescence imaging and found that the VPS35 D620N mutation induced endosomal dysfunction.

## Results

### Generation and characterization of iPSCs from PD patients and Controls

Analysis of induced neurons differentiated from iPSCs (iNeurons) enables the construction of pathological models using the patient’s own cells. Such analyses are particularly useful for the study of neurodegenerative disorders, because it is difficult to collect brain tissue samples from these patients.

First, we generated iPSCs from the peripheral blood mononuclear cells of two PD patients carrying the D620N mutation in the *VPS35* gene (PD1 and PD2) and two healthy controls (Ctrl1 and Ctrl2). PD1 and PD2 are familial PD patients from the same family, as described previously (Family A in [12]). A detailed characterization of the PD and control lines used in this study is illustrated in (Additional file 1: Table S1). Two of the control iPSC lines (Ctrl2-1, Ctrl2-2) have been characterized and published previously [38]. All iPSC lines were stained for pluripotency markers (NANOG and SSEA4) (Additional file 2: Figure S1a). These iPSCs were able to differentiate into cells of all three germ layers in vitro (DIV) (Additional file 2: Figure S1a) and had a normal karyotype (Additional file 2: Figure S1b).

### Differentiation and characterization of DA neurons

**Fig. 1:**
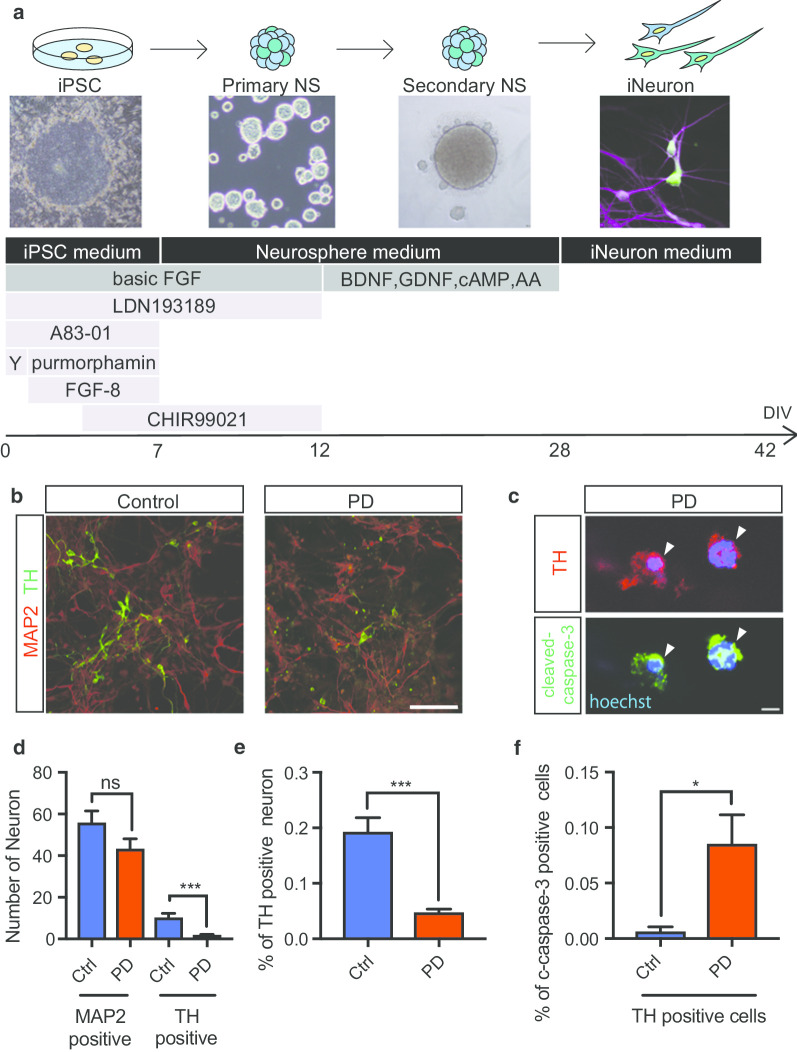
Differentiation and Characterization of iPSC-derived Dopaminergic (DA) Neurons. **a** Schematic overview of the conditions used to differentiate iPSCs into DA neurons, with bright-field representations of the cells at several stages of differentiation shown. **b** Immunostaining of differentiated DA neurons at 42 DIV. The neuronal marker MAP2 (red) and the DA neuron marker TH (green) are shown in the control and PD groups. **c** Immunostaining of apoptotic DA neurons (arrow heads) using markers of apoptosis (cleaved caspase-3) and DA neurons (TH). DA neurons were positive for both cleaved caspase-3 and TH. **d**, **e** Quantitative analysis of the number in a single field and percent of DA neurons among iPSC-derived neurons (iNeurons). The number and percent of TH-positive cells were lower in the PD group than in the control group (1.0 = 100%, n = 5 in control group, n = 5 in the PD group). **f** Quantitative analysis of the percent of apoptotic DA neurons among iNeurons. The percent of apoptotic cells was higher in the PD group than in the control group (1.0 = 100%, n = 3 in the control group, n = 3 in the PD group). Data are represented as mean ± SEM; n.s., not significant. *P < 0.05, ***P < 0.001; Mann–Whitney U-test in **d**–**f**. Scale bars, 100 μm in **b** and 5 μm in **c**. See also Additional file 1: Table S1, Additional file 2: Figure S1 and Additional file 3: Figure S2

PD is primarily a movement disorder and shows a predilection for nigral DA neurons. To study the effect of the VPS35 D620N mutation in the context of PD, iPSCs were differentiated into DA neurons (Fig. 1a). A total of six different iPSC clonal lines from two control individuals and two PD patients were differentiated into DA neurons as described previously [39] with minor modifications (Fig. 1a). Briefly, neural stem cells prepared from iPSCs were cultured in the presence of LND193189 and A83-01 to initiate neuronal induction with CHIR99021, FGF8, and purmorphamine. After 12 days in vitro (DIV), the cells were replated for differentiation into DA neurons with ascorbic acid, cyclic adenosine monophosphate (cAMP), brain-derived neurotrophic factor (BDNF), and glial cell line-derived neurotrophic factor (GDNF) for 18 days. All lines successfully differentiated into DA neurons (Fig. 1b). In this experiment, 47.9% of iPSC-derived cells were MAP2 positive neurons with long processes and 47% were GFAP positive glial cells with large cytoplasm (Additional file 3: Figure S2a, b, c).

The differentiation efficiency was assessed by determining expression of MAP2 and the DA neuronal marker tyrosine hydroxylase (TH) using immunofluorescence. At 42 DIV, most of the cells from healthy controls were positive for the neuronal marker MAP2, and approximately 20% of MAP2-positive cells were also positive for TH. In contrast, among the cells from PD patients, fewer than 5% of MAP2-positive cells were positive for TH, indicating the possibilities of low efficiency of dopaminergic differentiation and occurrence of cell death in PD-derived DA neurons (Fig. 1d, e). Next, to investigate the cause of this reduction in TH-positive cells in PD, we examined the expression of cleaved caspase-3 (an apoptotic marker) (Fig. 1c). The number of cleaved caspase-3-positive cells was increased in both PD1 and PD2 iPSC-derived DA neurons compared with healthy control iPSC-derived DA neurons (Fig. 1f). There was no significant difference in the number of cleaved caspase-3-positive cells in MAP2 positive neurons (Additional file 3: Figure S2d). These results indicate that DA neurons derived from PD patients carrying the VPS35 D620N mutation undergo apoptosis.

### Colocalization of Retromers with either Rab5a or Rab7a in endosomal vesicles

Endosomes carry a range of proteins for targeted delivery [30, 32, 40]. In the endosomal pathway, cargos are internalized from the cell surface, which regulates their storage and recycling, or sent to lysosomes for degradation [41]. Two of the primary players of this endosomal system are early and late endosomes, which can be distinguished by their associated Rab GTPases [42]; Rab5 coordinates clathrin-dependent endocytosis and the biogenesis of early endosomes and their fusion, whereas Rab7 regulates the transport and maturation of acidic late endosomes as well as their fusion with lysosomes [33]. Rab conversion, in which Rab7 supplants Rab5, is a key event in endosome maturation. The levels of both endosomal Rab5 and Rab7 vary during endosome maturation. The retromer complex is a key player in the endosomal trafficking of proteins and sorting. A past study suggested that the retromer is active during endosome maturation and that Rab7a mediates recruitment of the cargo-selective retromer complex [17].

**Fig. 2:**
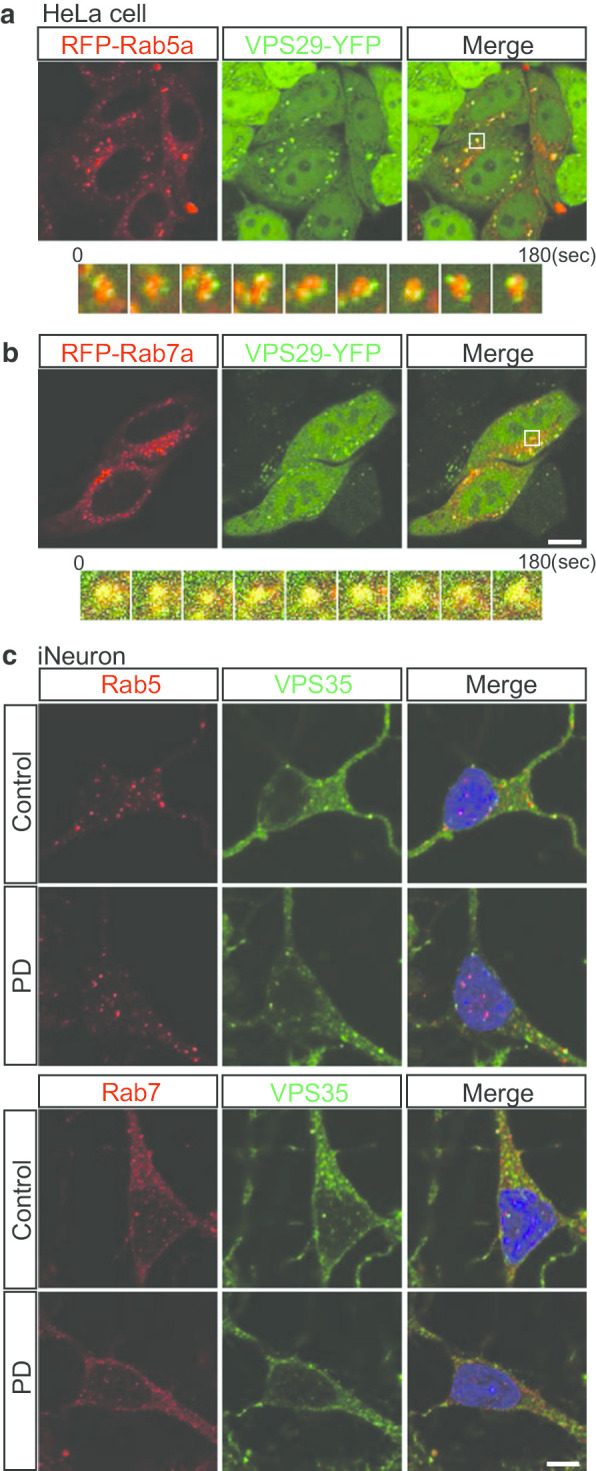
Colocalization of Retromers with Either Early or Late Endosomes. **a**, **b** HeLa cells cotransfected with fluorescently labeled VPS29-YFP (green; retromers) and either RFP-Rab5a (red; early endosomes) or RFP-Rab7a (red; late endosomes) were imaged by time-lapse fluorescence microscopy. The pictures in this figure were extracted from Additional file 5: Movies S1 and Additional file 7: Movie S3. Vesicles positive for VPS29 were almost always found adjacent to Rab5a-positive vesicles. VPS29-positive vesicles were almost always double positive for Rab7a-positive vesicles. Yellow indicates the overlapping localization of green and red signals. **c** Immunostaining of iPSC-derived neurons (iNeurons) for endogenous VPS35 and either Rab5 or Rab7; Rab5 and Rab7 partially colocalized with VPS35. There was no significant difference between the PD and control groups. Scale bars, 10 μm in **a** and **b** and 5 μm in **c**. See also Additional file 4: Figure S3 and Additional file 5: Movies S1, Additional file 6: Movie S2, Additional file 7: Movie S3, Additional file 8: Movie S4

Since the retromer complex plays an important role in endosomal trafficking, we used live-cell imaging technique to observe retromers and endosomes as previously described [21, 34]. To visualize movement of the retromer complex, we labeled endogenous retromers by the transduction of fluorescently labeled VPS29 (VPS29-YFP), a component of the retromer complex. Most of the VPS29 in HeLa cells seems to be incorporated into retromers, since 98.9% of VPS29 positive vesicles were double positive for VPS35, a component of the retromer (Additional file 4: Figure S3a), although VPS29 is also known as a component of the retriever complex [18, 43]. Similar observation was obtained in MAP2 positive iNeurons as well (Additional file 4: Figure S3b). So that we use VPS29-YFP as a reporter of retromers. To determine whether the retromer complex is associated with endosomes, we used RFP-Rab5a and RFP-Rab7a, which label early and late endosomes, respectively [33]. By expressing RFP-Rab5a and RFP-Rab7a with VPS29-YFP, we found that the movement of VPS29-YFP (retromers) was often associated with both early and late endosome reporters in the cytoplasm of HeLa cells (Fig. 2a, b). Vesicles positive for VPS29 were almost always found adjacent to Rab5a-positive vesicles, in more detail, several VPS29-positive small vesicles moved around the Rab5a-positive vesicles without leaving the surface of endosomes. (Fig. 2a, Additional file 5: Movies S1 and Additional file 6: Movie S2). VPS29-positive vesicles were almost always double positive for Rab7a-positive vesicles and moving together as same vesicles (Fig. 2b, Additional file 7: Movies S3). Importantly, almost all retromer complexes moved with Rab5a- or Rab7a-positive vesicles. The extent to which VPS29 was co-labeled with the markers of Golgi, lysosome, and mitochondria was lower than that with endosomes, and dynamic coimaging showed that the retromer moved independent of lysosome (Additional file 8: Movie S4), the Golgi (data not shown), and mitochondria (data not shown).

Next, we performed immunocytochemistry against the known retromer component VPS35 in iNeurons. Many of Rab5 and Rab7 colocalized with VPS35 as observed in the live-cell imaging (Fig. 2c). However, there was no significant difference in this colocalization between the PD and control groups.

### VPS35 mutation affects the movement of early and late endosomes

**Fig. 3:**
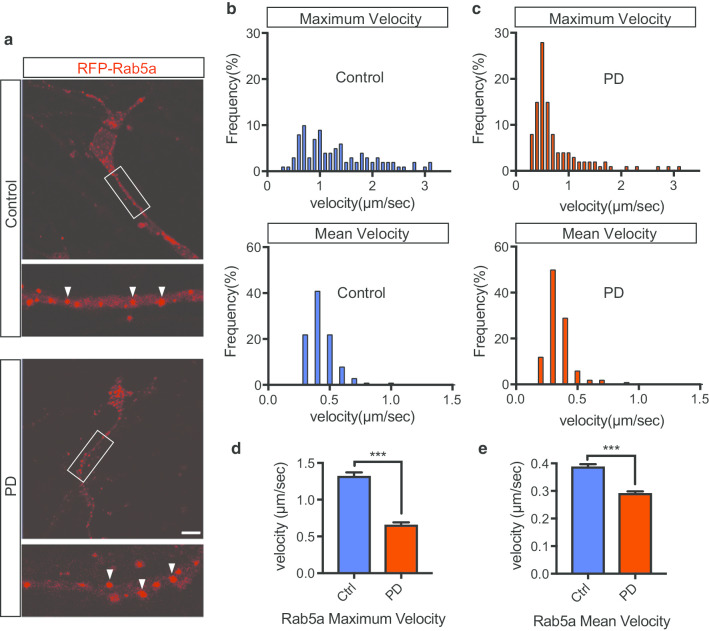
VPS35 Mutation Affects the Movement of Early Endosomes. **a** iPSC-derived neurons (iNeurons) stably expressing RFP-Rab5a were imaged by time-lapse fluorescence microscopy. RFP-Rab5a (red; early endosomes). Early endosomal vesicles are indicated by white arrows. **b** Distribution of the maximum velocities of moving RFP-Rab5a vesicles in the control and PD groups (n = 3 per line, total n = 200 moving vesicles in 20 neurons in each group). **c** Distribution of the mean velocities of moving RFP-Rab5a vesicles in the control and PD groups (n = 3 per line, total n = 200 moving vesicles in 20 neurons in each group). (d, e) Quantification of the maximum and mean velocities of vesicles (RFP-Rab5a) in iNeurons. Data are represented as mean ± SEM; ***P < 0.001; Mann–Whitney U-test in **d** and **e**. Scale bar, 10 μm in **a**. See also Additional file 9: Movies S5 and Additional file 10: Movie S6

To assess the effect of VPS35 mutation on endosomal trafficking, we first visualized the trafficking of early endosomes in iNeurons by live-cell imaging. To this end, we transduced cultured iNeurons (DIV42) with RFP-Rab5a and simultaneously visualized their trafficking in neurites for one minute (Fig. 3a, Additional file 9: Movie S5, Additional file 10: Movie S6). In this experiment, cells with processes less than 4 μm width and longer than 10 μm length were considered as iNeurons and used for the analysis (Additional file 3: Figure S2b). Single-particle tracking analysis revealed the presence of Rab5a-positive vesicles in static and fast-moving states; some Rab5a-positive vesicles were static, while others moved quickly in the anterograde and retrograde directions in neurites. Velocity histograms of mobile Rab5a-positive vesicles are shown as kymographs (Fig. 3b, c). The maximum and mean velocities of early endosomes in the PD group were lower than those in the control group (Fig. 3d, e). The average speed of individual endosomes differed between the control and PD groups, suggesting that static Rab5a-positive vesicles are increased in PD.

**Fig. 4:**
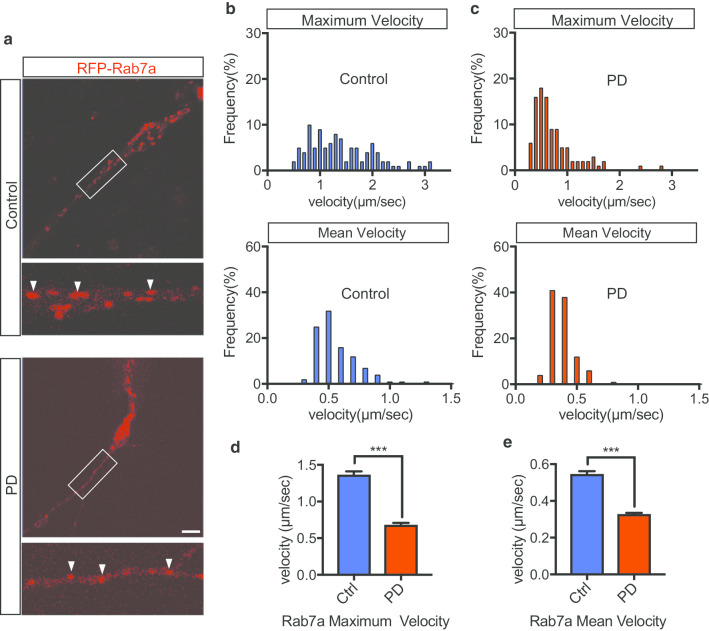
VPS35 Mutation Affects the Movement of Late Endosomes**.** **a** iPSC-derived neurons (iNeurons) stably expressing RFP-Rab7a were imaged by time-lapse fluorescence microscopy. RFP-Rab7a (red; late endosomes). Late endosomal vesicles are indicated by white arrows. **b** Distribution of the maximum velocities of moving RFP-Rab7a vesicles in the control and PD groups (n = 3 per line, total n = 140 moving vesicles in 14 neurons in each group). **c** Distribution of the mean velocities of moving RFP-Rab7a vesicles in the control and PD groups (n = 3 per line, total n = 140 moving vesicles in 14 neurons in each group). **d**, **e** Quantification of the maximum and mean velocities of vesicles (RFP-Rab7a) in iNeurons. Data are represented as mean ± SEM; ***P < 0.001; Mann–Whitney U-test in **d** and **e**. Scale bar, 10 μm in **a**. See also Additional file 11: Movie S7 and Additional file 12: Movie S8

Next, we visualized the trafficking of late endosomes in iNeurons. We transduced cultured iNeurons (DIV42) with RFP-Rab7a and visualized their trafficking in neurites for one minute (Fig. 4a, Additional file 11: Movie S7, Additional file 12: Movie S8). Similar to Rab5a-positive vesicles, some Rab7a-positive vesicles move quickly in the anterograde and retrograde directions in neurites. Interestingly, many of the moving early and late endosome (Rab5a: Control 80%, PD 76.7%. Rab7a: Control 70%, PD 63.3%) change a direction at least once from anterograde to retrograde or retrograde to anterograde (Additional file 13: Figure S4). Velocity histograms of mobile Rab7a-positive vesicles are shown as kymographs (Fig. 4b, c). The maximum and mean velocities of Rab7a-positive vesicles in the PD group were lower than those in the control group (Fig. 4d, e). The average speed of individual endosomes differed between the control and PD groups. These results indicate that VPS35 mutation affects the movement of early and late endosomes.

### VPS35 mutation causes endosomal fission and fusion dysfunction

In processes during the endosomal trafficking of vesicles, such as sorting, tubulation, and fission/fusion events, the retromer and WASH complex are activated through their direct interaction, leading to the formation of tubular structures, and the WASH complex promotes the fission of tubular structures [44, 45]. A mutation in VPS35 (D620N) diminishes the interaction between the WASH complex and retromer as well as impairs the fission process [18, 46].

**Fig. 5:**
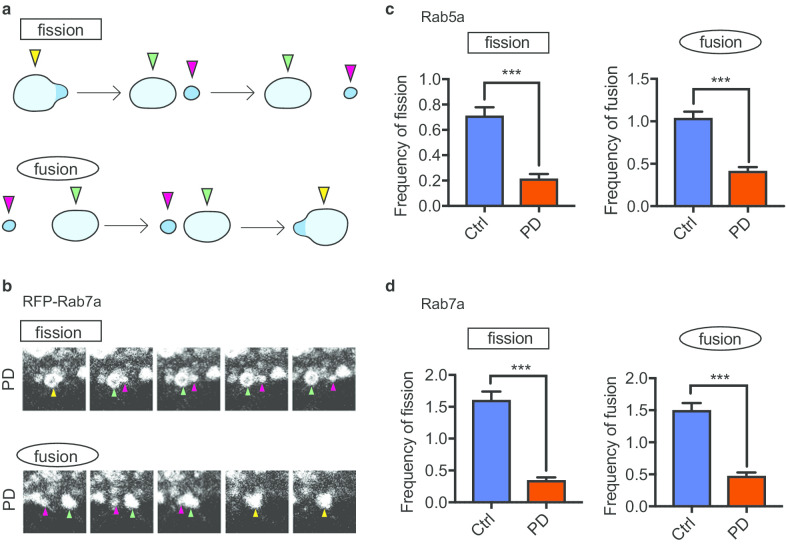
VPS35 Mutation Causes Defects of Endosomal Fission and Fusion. **a** Schematic overview of endosomal fission and fusion. **b** The fission and fusion of RFP-Rab7a vesicles in the neurites of iNeurons in the PD group were imaged by time-lapse fluorescence microscopy. **c** Quantitative analysis of the frequency of fission and fusion of RFP-Rab5a vesicles. The frequency of vesicular fission was lower in the PD group than in the control group. Similarly, the frequency of vesicular fusion was lower in the PD group than in the control group (n = 3 per line, total n = 200 moving vesicles in 20 neurons in each group). **d** Quantitative analysis of the frequency of fission and fusion of RFP-Rab7a positive vesicles. The frequency of vesicular fission was lower in the PD group than in the control group. Similarly, the frequency of vesicular fusion in the PD group than in the control group (n = 3 per line, total n = 140 moving vesicles in 14 neurons in each group). Data are represented as mean ± SEM; ***P < 0.001; Mann–Whitney U-test in **c** and **d**. See also Additional file 9: Movies S5, Additional file 10: Movie S6, Additional file 11: Movie S7, Additional file 12: Movie S8, and Additional file 14: Figure S5

To assess the effect of VPS35 mutation on endosomal trafficking, especially the processes of fission and fusion, we next investigated the efficiency of vesicular fission and fusion in the endosome. We counted vesicles in iNeurons that underwent fission and fusion for one minute. We collected time-lapse imaging data from iNeurons transfected with RFP-Rab5a and RFP-Rab7a in the same manner, as shown in Figs. 3 and 4, and observed the movement of individual vesicles in endosomes and counted fission and fusion events for one minute (Fig. 5a, b, Additional file 14: Figure S5). The fission frequency for both Rab5a- and Rab7a-positive vesicles was significantly lower in the PD group than in the control group (Fig. 5c, d). Similarly, the fusion frequency for both Rab5a- and Rab7a-positive vesicles was lower in the PD group than in the control group (Fig. 5c, d). Therefore, our results demonstrate that VPS35 mutation causes endosomal fission and fusion dysfunction.

### VPS35 mutation causes CI-MPR transport defects in glial cells differentiated from iPSCs

**Fig. 6:**
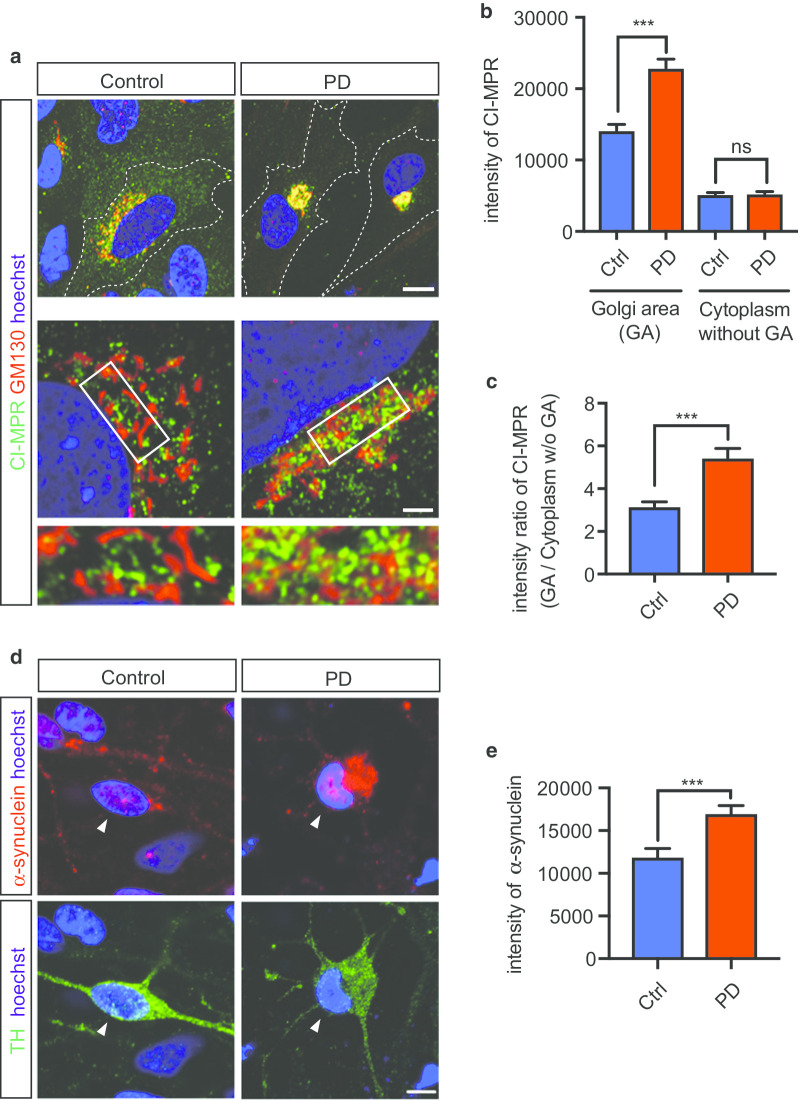
Distribution of CI-MPR in Glial Cells Derived from iPSCs. **a** Immunostaining of glial cells for CI-MPR and the Golgi. A proportion of CI-MPR was localized to the perinucleus around the Golgi (Golgi area) in the control group. In the PD group, CI-MPR appeared to accumulate around the Golgi. **b** Quantification of the results of localization analysis performed in **a**. The figure shows the intensity of CI-MPR in the cytoplasm (without Golgi area) and Golgi area (n = 3 per line, total n = 69 cells in each group). **c** Quantification of the results of localization analysis performed in **a** using the ratio of intensities in the Golgi/cytoplasm (without Golgi) (n = 3 per line, total n = 69 cells in each group). **d** Immunostaining of iPSC-derived DA neurons (arrow heads) for α-synuclein and TH. **e** Quantification of the intensity of α-synuclein in the cytoplasm in iPSC-derived DA neurons. The intensity of α-synuclein in the cytoplasm was higher in the PD group than in the control group. (n = 3 per line, total n = 45 cells in Control, n = 39 cells in PD). Data are represented as mean ± SEM; ***P < 0.001; Mann–Whitney U-test in **b** and **c**. Scale bars, 10 μm in the upper part of **a**, 2 μm in the lower part of **a** and 5 μm in **d**. See also Additional file 15: Figure S6

Several retromer cargo proteins, such as CI-MPR, are essential for the delivery of the main component enzymes of lysosomes. One of the best-characterized cargos of the retromer is CI-MPR, which participates in the delivery of lysosomal enzymes, such as the aspartyl protease cathepsin D, to lysosomes [21]. Cathepsin D is the primary lysosomal enzyme that degrades α-synuclein, the etiologic protein of PD [47, 48]. Numerous past studies have used CI-MPR to assay retromers [19–21]. Next, to assess the effects of VPS35 mutation on endosomal trafficking and the localization of cargo proteins of retromers, we examined the localization of endogenous CI-MPR in glia from PD patients and healthy controls (Fig. 6a). In this experiment, cells with high ratio (more than 2.5) of total cell area (cytoplasm plus nucleus)-to-nucleus area were considered as glial cells for the analysis, since GFAP positive glial cells can be easily distinguished from iNeuron according to this criteria (Additional file 3: Figure S2b, c, Additional file 15: Figure S6a, c). CI-MPR was localized to the perinucleus around the Golgi in the control group. In the PD group, CI-MPR appeared to accumulate around the Golgi (Fig. 6b). The intracellular distribution of endogenous CI-MPR was assessed by determining the ratio of the CI-MPR intensity in the Golgi to that in the cytoplasm, and the ratio was greater in the PD group than in the control group (Fig. 6c). Analysis of the CI-MPR distribution in iNeuron was difficult to detect, because the area of the Golgi in iNeuron was too small to analyze; however, quantification of the intensity of CI-MPR staining in neurites reveled that there was no difference between PD and Control groups (Additional file 15: Figure S6a, b).

### VPS35 mutation causes accumulation of α-synuclein in DA neurons differentiated from PD-derived iPSCs

Pathological feature of PD is accumulation of α-synuclein in the Lewy body [4]. We examined α-synuclein levels in cytoplasm of TH positive neuron differentiated from iPSCs. Immunostaining analysis showed an increase in intracellular α-synuclein intensity in DA neurons derived from PD patients (Fig. 6d, e). These results demonstrate a possibility of PD-relevant neuropathological feature in DA neurons derived from PD patients carrying the VPS35 D620N mutation.

## Discussion

In the current study, we show that the VPS35 mutation (D620N) decreased the velocity and deficits in fission and fusion in endosomes from iNeurons.

VPS35, a major component of the retromer complex, is a key player in endosomal trafficking and the recognition of cargo proteins. Past studies have shown that the retromer has diverse roles in the endosomal system, such as trafficking, sorting, tubulation, and fission [14, 30]. Furthermore, our findings provide support for the results of these past studies. VPS35 is structurally important in the retromer complex; for example, VPS35 connects to sorting nexins (SNXs) through VPS26, inducing endosomal tubulation. VPS35 directly contacts FAM21, a part of the WASH complex, which leads to vesicle fission in endosomes [44, 45, 49–51]. We suggest that the VPS35 D620N mutation may change the three-dimensional structure of VPS35 and affects the functions of the retromer, such as endosomal tubulation and fission, slowing the movement of early and late endosomes.

In the process of endosomal fission, first, the retromer recruits the WASH complex through their direct interaction in endosomes. WASH plays a major role in the polymerization of endosomal actin [44], which promotes the formation of retromer tubules. WASH functions to assist the fission of tubular structures in endosomes [44, 45, 52]. A previous study showed that the VPS35 mutation impairs the association and recruitment of the WASH complex to endosomes [46]. Our data support these data, and in the current study, we have shown for the first time that the VPS35 mutation impairs endosome fission in iNeurons.

The fusion of early endosomes requires Rab5 and COVERT, a multiprotein complex [52, 53]. However, in a previous study, there was no evidence that retromers are involved in the process of endosomal fusion. In the current study, our data strongly suggests that the VPS35 mutation directly or indirectly impairs the endosomal fusion system, similar to its effects on fission. Since the VPS35 mutation impaired the fusion of endosomes, the retromer may have an unknown role in regulating fusion as well.

A number of studies have demonstrated the association between retromer and endosomes. Seaman et al. found a significant colocalization between VPS26 and Rab5 and modest colocalization between VPS26 and Rab7 in HeLa cells [21]. On the other hand, Rojas discovered that VPS29 is almost always found on domains of the endosomal vesicles contained Rab7 and VPS29 is also found in association with endosomal vesicles contained Rab5a, but VPS29 and Rab5a are largely segregated to different domains [34]. Our live-imaging show the appearance of VPS29 attaching and moving together with early and late endosomal vesicles which are dynamically moving around in cytoplasm of HeLa cells. These results indicate the close relationship between retromer and endosomes as previous studies.

Several retromer cargo proteins, such as CI-MPR, are essential for delivery of the main component enzymes of lysosomes. Retromer dysfunction, therefore, disrupts lysosomal function and integrity. It has also been reported that lysosomes and the lysosomal enzyme cathepsin D are fundamental regulators of α-synuclein degradation through the chaperone-mediated autophagy pathway [47, 48].

CI-MPR is found in the TGN, early endosomes, late endosomes, and the plasma membrane [54]. CI-MPR is primarily present in the TGN and transported between the TGN and endosomes. One of the most widely accepted tenets of retromer function is that the retromer complex mediates the endosome-to-Golgi retrieval of CI-MPR [19, 21]. However, recently published data have questioned the validity of this long-established theory [55]. Two studies indicated that the SNX-BAR dimer associate with the CI-MPR to mediate its retrieval independent of the retromer [56, 57].

There are several conflicted research findings about CI-MPR trafficking. Overexpression of VPS35 D620N or shRNA for VPS35 reduces CI-MPR colocalization with Golgi in primary rat neurons [58]. Similar results are obtained in patient-derived VPS35 D620N fibroblasts [59]. In contrast, Follett et al. demonstrate that CI-MPR localizes on perinuclear in A431 cells transfected with VPS35 D620N as well as in PD patient-derived fibroblasts with VPS35 D620N [60]. Fuse et al. demonstrate CI-MPR colocalized with Golgi in the VPS35 knockdown HeLa cells [61], sharing similar results with our findings. On the other hand, another study reports VPS35 D620N expressing HeLa cells exhibit normal CI-MPR localization [46].

Interestingly, our study revealed that endogenous CI-MPR appeared to accumulate around the TGN in glia cells differentiated from iPSCs of PD patients. CI-MPR-containing tubule-vesicular carriers could directly fuse with endosomes from the TGN to deliver their cargo. CI-MPR binds the cargo in the TGN and is then packaged into transport carriers that deliver the receptor with its bound ligand to early endosomes [54, 62]. Our results suggest that CI-MPR trafficking from the TGN to endosomes may be impaired in patient cells. In addition, our study showed that the retromer may directly or indirectly regulate the trafficking of CI-MPR, indicating that the VPS35 mutation impairs the trafficking of CI-MPR from the TGN to early endosomes.

VPS35 and retromer dysfunction are also directly connected to the pathological effects of α-synuclein, as the loss of VPS35 function sensitized cells to the accumulation of α-synuclein by interfering with the degradation machinery in a range of model systems [63–66]. In patient-derived fibroblasts as well as in Drosophila cells, VPS35 depletion caused α-synuclein accumulation [60, 64]. A functional interaction of VPS35 and α-synuclein has been reported previously, demonstrating the exacerbation of α-synuclein pathobiology by VPS35 deletion in mouse hippocampus [63] and α-synuclein accumulation in ventral midbrain of mice with VPS35 depletion [65, 66]. In contrast, recent study showed the progressive degeneration of dopaminergic neurons without evidence of α-synuclein positive neuropathology in ventral midbrain of aged VPS35 D620N knockin mice [67]. Our results demonstrate the induction of cell death and α-synuclein accumulation in DA neurons derived from PD patients carrying the VPS35 D620N mutation. Therefore, PARK17 patient-derived iPSCs provide an excellent experimental tool for understanding the pathophysiology underlying PD.

Interestingly, reduced VPS35 levels predispose patients to Alzheimer’s pathology [68], and pharmacological chaperones that stabilize the retromer complex promote its function in APP trafficking [69], suggesting that similar approaches may be beneficial in PD. The dysfunction of VPS35/the retromer is believed to be a risk factor for the pathogenesis of both AD and PD. Furthermore, VPS35 deficiency enhanced AD neuropathology in a Tg2576 mouse model of AD [23]. A current report identified that VPS35 regulates tau phosphorylation and neuropathology in tauopathies, such as progressive supranuclear palsy (PSP) and Pick’s disease [70].

Our findings indicate that VPS35 regulates endosomal trafficking in neurons. We suggest VPS35 as a potential therapeutic target for PD, AD, and other neurodegenerative diseases.

## Methods

### Generation of iPSCs and cell culture

All PD iPSCs and control iPSCs were generated from human peripheral blood mononuclear cells using episomal vectors according to a protocol from the Centre for iPSC Cell Research and Application (Kyoto University, Japan). All iPSC lines were cultured on mouse feeder cells in iPSC medium, which consisted of primate ES cell medium (ReproCELL) containing 10 μg/ml of bFGF (Wako).

### Neural induction from human iPSCs

All iPSC lines were differentiated into DA neurons according to a protocol from the Centre for iPSC Cell Research and Application (Kyoto University, Japan) [39] with minor modifications. After passaging the iPSCs, we added LDN193189 (Stemgent) and A83-01 (Wako) to the iPSC medium to efficiently induce neuronal differentiation. We also added purmorphamine (Cayman Chemical) and FGF8 (Wako) beginning at 1 DIV and CHIR99021 (Cayman Chemical) beginning at 3 DIV. At 12 DIV, the cells were dissociated into single cells after 10 min of incubation with TrypLE Select (Gibco) and passaged in a flask by sphere formation, following which the medium was exchanged with neurosphere medium consisting of KBM neural stem cell medium (KOHJIN BIO) and B27 supplement (Gibco) containing 10 μm/ml bFGF, human LIF (Millipore), LDN192189, and CHIR99021 from 7 to 12 DIV. At 12 DIV, cells in neurospheres were dissociated into single cells after 10 min of incubation with TrypLE Select and replated on low-cell adhesion 96-well plates (Thermo) at a density of 5–8 × 10^4^ cells/well in neurosphere medium containing 10 ng/ml GDNF, 200 mM ascorbic acid, 20 ng/ml BDNF (all Wako), and 400 μM dbcAMP (Sigma-Aldrich). Subsequently, we exchanged the medium every 2–3 days. At 28 DIV, cells in neurospheres were dissociated into single cells after 10 min of incubation with Accutase (Innovative Cell Technologies) and plated on glass dishes coated with poly-L-lysine (Sigma-Aldrich) and laminin (Gibco) with iNeuron medium consisting of neural differentiated media (Wako) until 42 DIV.

### Cell culture

HeLa cells were grown in Dulbecco’s Modified Eagle’s medium (Invitrogen) supplemented with 10% fetal bovine serum (FBS) and 1% penicillin–streptomycin in 5% CO_2_ in a humid incubator at 37 °C.

### Immunofluorescence studies

For immunocytochemical analysis, cells were fixed with 4% paraformaldehyde for 5 min (Fig. 1) or 10% trichloroacetic acid for 10 min (Figs. 2c, 6a, Additional file 4: Figure S3 and Additional file 15: Figure S6) at room temperature. After permeabilization and blocking with 0.3% Triton X-100 for 30 min and 5% FBS for 90 min, the cells were incubated with primary antibodies at 4 °C overnight. Primary antibodies against the following were used for these analyses: Nanog (rabbit, 1:200; ReproCELL), SEEA-4 (mouse, 1:200; Millipore), αSMA (mouse, 1:50; Dako), SOX-17 (goat, 15 μg/ml; R&D Systems), MAP2 (mouse, 1:1000; Sigma-Aldrich), MAP2 (chicken, 1:10,000; Abcam), MAP2 (rabbit, 1:1000; Abcam), TH (rabbit, 1:500; Millipore), TH (mouse, 1:500; Millipore), cleaved caspase-3 (rabbit, 1:200; Cell Signaling), VPS35 (goat, 1:300; Abcam), Rab5 (rabbit, 1:300; Abcam), Rab7 (mouse, 1:1000; Abcam), CI-MPR (mouse, 1:100; Abcam), GM130 (rabbit, 1:100; Abcam), α-synuclein (rabbit, 1:150; Cell Signaling), GFAP (chicken, 1:1000; Abcam) and VPS29 (rabbit, 1:50; Sigma-Aldrich). The following day, the cells were washed two times with PBS and incubated with secondary antibodies for 60 min at room temperature. The secondary antibodies used were goat or donkey antibodies conjugated to Alexa 488, 546, 633, or 647 (1:500; Invitrogen). Nuclear staining was performed with Hoechst solution (1:10,000; Invitrogen) with secondary antibodies. The immunoreactive cells were visualized using a confocal laser microscope (LSM880; Carl Zeiss). The length of cell processes and areas of the cell body and nucleus were quantified using ZEN software (Carl Zeiss).

To quantify the intensity of CI-MPR and α-synuclein shown in Fig. 6, images were analyzed by ZEN software (Carl Zeiss). Briefly, Immunostaining images (Fig. 6b,c and e) were taken by same pinhole, digital offset and master gain using LSM880 (Carl Zeiss). Areas of cytoplasm, cytoplasm without Golgi and Golgi were measured using ZEN software. The intensity ratio was calculated by measuring the intensity of CI-MPR in the Golgi area and cytoplasm without Golgi area.

### Cell transduction and time-lapse fluorescence microscopy

HeLa cells and iNeurons (iPSC-derived induced neurons) were transduced with lentivirus encoding VPS29-YFP or baculovirus encoding Rab5a or Rab7a fused with RFP at N-terminal (Life Technologies CellLight Reagents BacMam 2.0; C10587 and C10589, respectively). Lentivirus expression plasmids were constructed by inserting the VPS29-YFP fragment into the CSII-CMV-MCS vector from RIKEN BioResource Research Center. VPS29 and YFP were conjugated by PCR. Lentiviral vectors were generated according to a lentiviral vector preparation protocol from RIKEN BioResource Research Center. Sixteen to twenty-four hours after transduction, cells were imaged at 37 °C in a stage incubator (Carl Zeiss), and time-lapse fluorescence images were acquired with a confocal laser microscope (LSM880, Carl Zeiss). Cells with long neurites were chosen as iNeurons for the experiments. Images of vesicles were captured, and data acquisition was performed using Imaris software (Carl Zeiss). Baculovirus (Life Technologies CellLight Reagents BacMam 2.0; C10597) transduction was used to visualize markers of lysosomes.

### Statistical analyses

Statistical analyses of the obtained data were performed using Mann–Whitney U-test (*P < 0.05, **P < 0.01, ***P < 0.001), and the mean and standard error of the mean were plotted using Prism (Max OS X). The number of independent experiments (n) is indicated in each figure legend. Experimental data from each cell lines are presented separately in Additional file 16: Figure S7.

## Supplementary information

**Additional file 1: Table S1. **Summary of the iPSCs used in this study. iPSC lines were derived from healthy controls and patients with Parkinson’s disease and the* VPS35* D620N mutation (PARK17). All cells were obtained from peripheral blood mononuclear cells.

**Additional file 2: Figure S1. **Generation and characterization of iPSCs from PD (PARK17) patients and healthy controls. (a) Cell morphology and expression of human embryonic stem cell markers. iPSCs were obtained from healthy controls and PD patients with the *VPS35* D620N mutation. Control and PD patient iPSCs were morphologically identical to human embryonic stem cells (ESCs) and expressed the pluripotent stem cell markers NANOG and SSEA4. Nuclei were stained with Hoechst. *In vitro* differentiation of iPSCs to three germ layers identified by the following markers: Sox17 (endoderm), αSMA (mesoderm), and MAP2 (ectoderm). (b) Karyotype analysis of control and PD patient iPSCs. Scale bars, 100 μm in (a).

**Additional file 3: Figure S2.** Efficient of neural induction, morphological analysis and cell death. (a) ratio of MAP2 positive neuron and GFAP positive glia. (b) length (micro meter) of cell processes of MAP2 positive neuron and GFAP positive glia. (c) ratio of nucleus / nucleus+cytoplasm. (d) Quantitative analysis of the percent of apoptotic neurons among iNeurons (MAP2 positive cells). The percent of apoptotic cells was higher in the PD group than in the control group, but there was no significant difference between the PD and control groups. (1.0 = 100%, n = 3 in the control group, n = 3 in the PD group). Data are represented as mean ± SEM; n.s., not significant. Mann-Whitney U-test in (d).

**Additional file 4: Figure S3.** Immunostaining of HeLa cells and iNeurons for endogenous VPS35 and VPS29. (a) Immunostaining of HeLa cells for endogenous VPS35 and VPS29. (b) Immunostaining of iNeuron for endogenous VPS35 and VPS29. Scale bars, 5 μm in (a) and (b).

**Additional file 5: Movie S1.** Colocalization of retromers (VPS29-YFP; Green) with early endosomes (RFP-Rab5a; Red) in HeLa cells.

**Additional file 6: Movie S2.** Magnified view showing the colocalization of retromers (VPS29-YFP; Green) with early endosomes (RFP-Rab5a; Red) in HeLa cells.

**Additional file 7: Movie S3.** Colocalization of retromers (VPS29-YFP; Green) with late endosomes (RFP-Rab7a; Red) in HeLa cells.

**Additional file 8: Movie S4.** Colocalization of retromers (VPS29-YFP; Green) with lysosomes (Lamp1-RFP; Red) in HeLa cells.

**Additional file 9: Movie S5.** iPSC-derived neurons (iNeurons) from healthy controls stably expressing RFP-Rab5a were imaged by time-lapse fluorescence microscopy.

**Additional file 10: Movie S6.** iPSC-derived neurons (iNeurons) from PD patients stably expressing RFP-Rab5a were imaged by time-lapse fluorescence microscopy.

**Additional file 11: Movie S7. ** iPSC-derived neurons (iNeurons) from healthy controls stably expressing RFP-Rab7a were imaged by time-lapse fluorescence microscopy.

**Additional file 12: Movie S8.** iPSC-derived neurons (iNeurons) from PD patients stably expressing RFP-Rab7a were imaged by time-lapse fluorescence microscopy.

**Additional file 13: Figure S4.** Movement of early and late endosome in neurites. (a) Movement of Rab5a positive early endosomes. (b) Movement of Rab7a positive late endosomes.

**Additional file 14: Figure S5.** Endosomal fission and fusion related to the data in Figure 5. (a) RFP-Rab5a in the neurites of control and PD iNeurons was imaged by time-lapse fluorescence microscopy. (b) RFP-Rab7a in the neurites of control iNeurons was imaged by time-lapse fluorescence microscopy.

**Additional file 15: Figure S6.** Localization of CI-MPR in neurons derived from iPSCs (iNeurons) related to the data in Figure 6. (a) Immunostaining of neurons derived from iPSCs (iNeurons) from PD patients and healthy controls for endogenous CI-MPR and the Golgi. Most CI-MPR was localized around the Golgi. (b) Quantification of the results of the localization analysis performed in (a). The figure shows the intensity of CI-MPR staining in neurites (n = 3 per line). There was no difference in intensity between PD and Control groups. (c) Immunostaining of glial cells derived from iPSCs from healthy controls for endogenous CI-MPR, GM130 and GFAP. Data are represented as mean ± SEM; n.s., not significant; Mann–Whitney U-test in (b). Scale bar, 10 μm in (a) 5 μm in (c).

**Additional file 16: Figure S7.** Experimental results in each iPS line.

## Data Availability

The datasets used and analyzed during the current study are available from the corresponding authors on reasonable request.

## Abbreviations

AD: Alzheimer’s disease
BDNF: Brain-derived neurotrophic factor
CI-MPR: Cation-independent mannose 6-phosphate receptor
DA neuron: Dopaminergic neuron
GDNF: Glial cell-derived neurotrophic factor
iPSCs: Induced pluripotent stem cells
PBS: Phosphate-buffered saline
PD: Parkinson’s disease
PFA: Paraformaldehyde
Rab5a: Ras-related protein Rab-5a
Rab7a: Ras-related protein Rab-7a
RFP: Red fluorescent protein
TH: Tyrosine hydroxylase
VPS35: Vacuolar protein sorting 35
YFP: Yellow fluorescent protein
