# Supplementary material for: Endosomal dysfunction in iPSC-derived neural cells from Parkinson’s disease patients with VPS35 D620N
Source: Mol Brain. 2020 Oct 8;13:137. doi: 10.1186/s13041-020-00675-5 (PMC7542911; doi:10.1186/s13041-020-00675-5)
Supplement: Supplementary file 1 — Additional file 1: Table S1. Summary of the iPSCs used in this study. iPSC lines were derived from healthy controls and patients with Parkinson’s disease and the VPS35 D620N mutation (PARK17). All cells were obtained from peripheral blood mononuclear cells. [file 13041_2020_675_MOESM1_ESM.docx]

| Additional File Table S1. Summary of the Healthy Controls and Patients Used in This Study | | | | |  |
| --- | --- | --- | --- | --- | --- |
|  | Age at Biopsy | Gender | Mutation | Status | Code  (iPSc line) |
| Control1 | 50s | M | - | Control | Ctrl1-1 |
| Control2 | 60s | M | - | Control | Ctrl2-1 |
|  |  |  |  |  | Ctrl2-2 |
| PD1 | 60s | M | *VPS35* p.D620N | Parkinson’s disease (PARK17) | PD1-1 |
|  |  |  |  |  | PD1-2 |
| PD2 | 70s | M | *VPS35* p.D620N | Parkinson’s disease (PARK17) | PD2-1 |
