# Supplementary figures and images for: Endosomal dysfunction in iPSC-derived neural cells from Parkinson’s disease patients with VPS35 D620N
Source: Mol Brain. 2020 Oct 8;13:137. doi: 10.1186/s13041-020-00675-5 (PMC7542911; doi:10.1186/s13041-020-00675-5)

**a**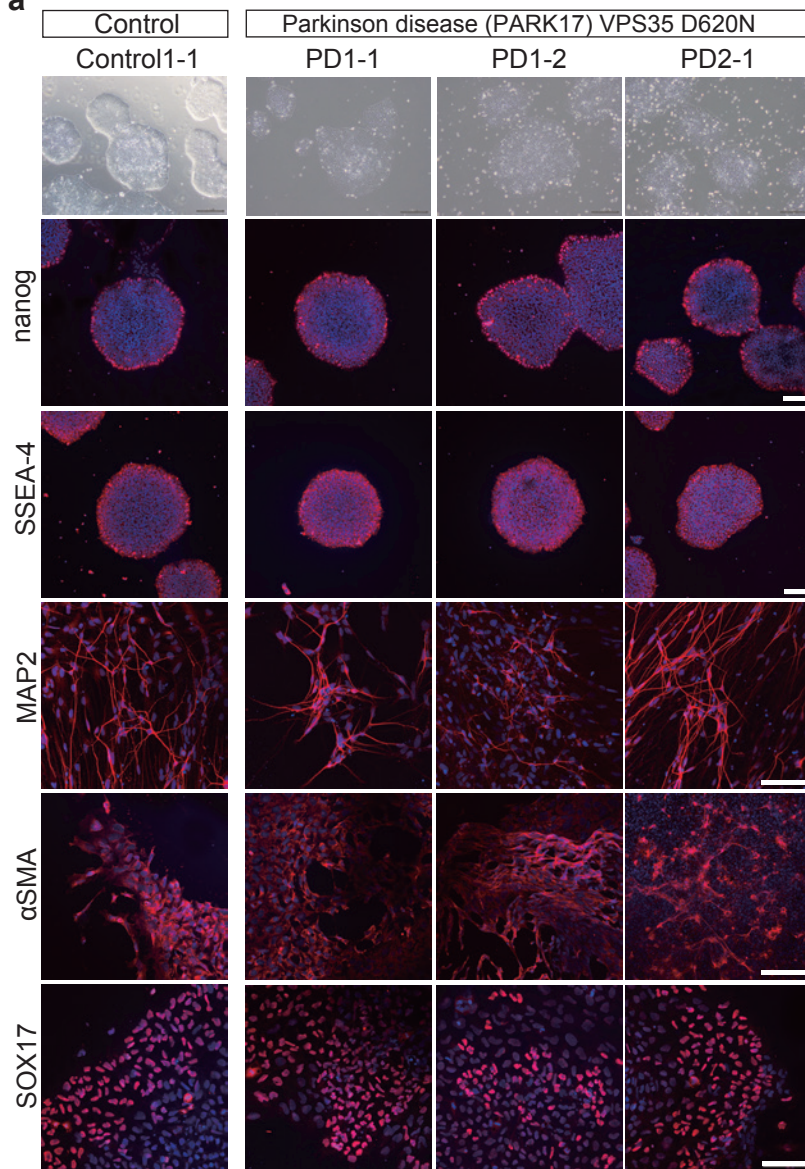**b**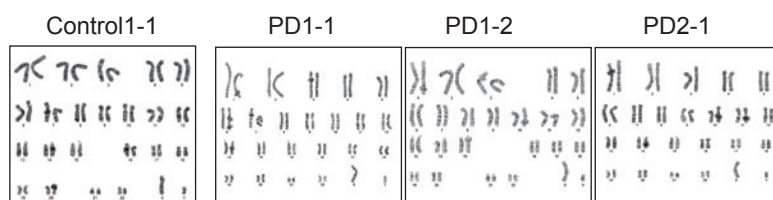

Supplement: Supplementary file 2 — Additional file 2: Figure S1. Generation and characterization of iPSCs from PD (PARK17) patients and healthy controls. (a) Cell morphology and expression of human embryonic stem cell markers. iPSCs were obtained from healthy controls and PD patients with the VPS35 D620N mutation. Control and PD patient iPSCs were morphologically identical to human embryonic stem cells (ESCs) and expressed the pluripotent stem cell markers NANOG and SSEA4. Nuclei were stained with Hoechst. In vitro differentiation of iPSCs to three germ layers identified by the following markers: Sox17 (endoderm), αSMA (mesoderm), and MAP2 (ectoderm). (b) Karyotype analysis of control and PD patient iPSCs. Scale bars, 100 μm in (a). [file 13041_2020_675_MOESM2_ESM.pdf]

**a**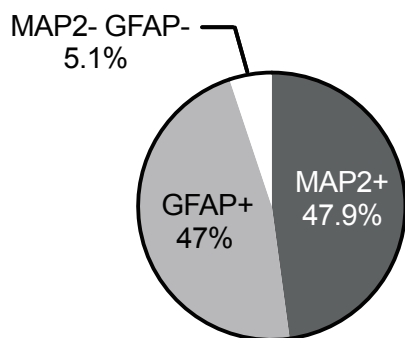**b**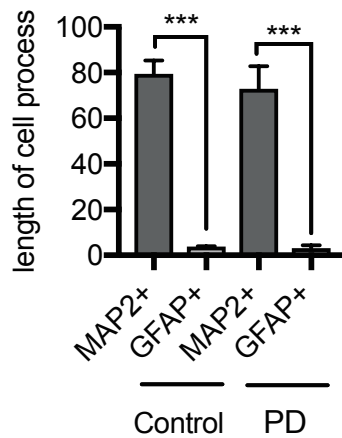**c**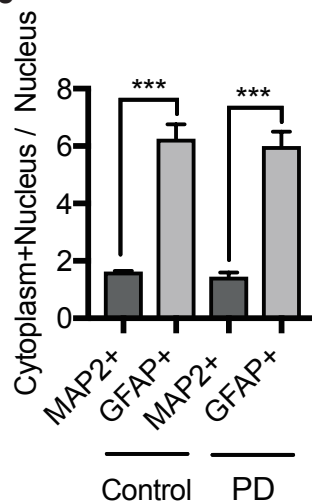**d**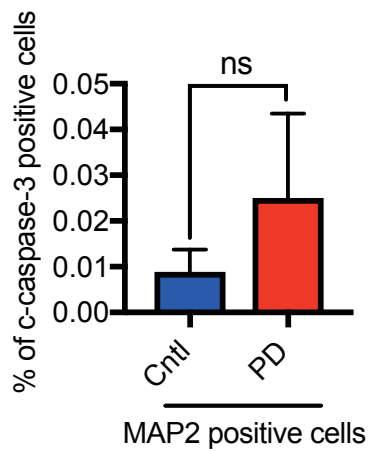

Supplement: Supplementary file 3 — Additional file 3: Figure S2. Efficient of neural induction, morphological analysis and cell death. (a) ratio of MAP2 positive neuron and GFAP positive glia. (b) length (micro meter) of cell processes of MAP2 positive neuron and GFAP positive glia. (c) ratio of nucleus / nucleus+cytoplasm. (d) Quantitative analysis of the percent of apoptotic neurons among iNeurons (MAP2 positive cells). The percent of apoptotic cells was higher in the PD group than in the control group, but there was no significant difference between the PD and control groups. (1.0 = 100%, n = 3 in the control group, n = 3 in the PD group). Data are represented as mean ± SEM; n.s., not significant. Mann-Whitney U-test in (d). [file 13041_2020_675_MOESM3_ESM.pdf]

**a**

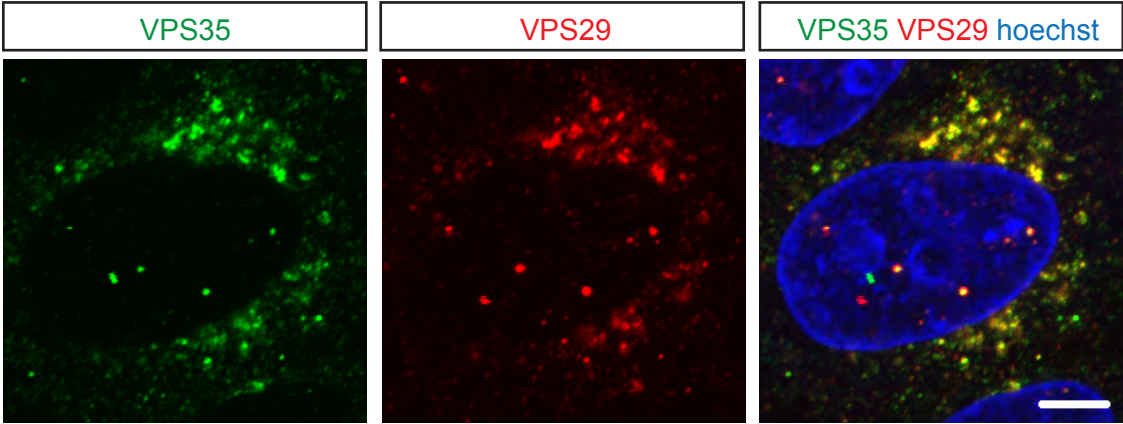

**b**

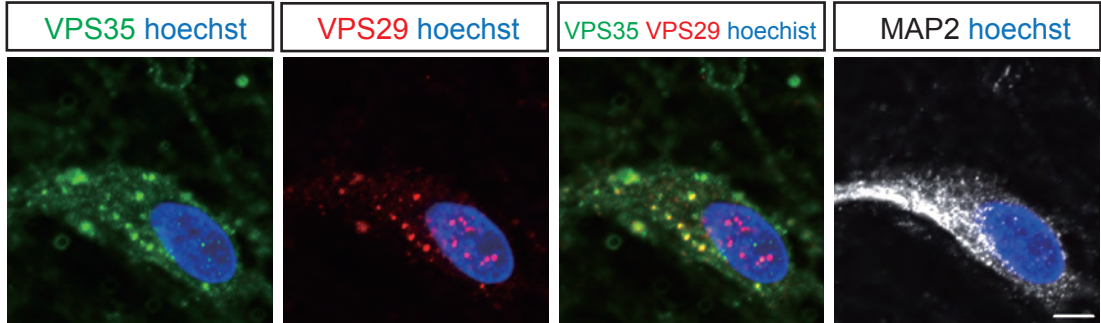

Supplement: Supplementary file 4 — Additional file 4: Figure S3. Immunostaining of HeLa cells and iNeurons for endogenous VPS35 and VPS29. (a) Immunostaining of HeLa cells for endogenous VPS35 and VPS29. (b) Immunostaining of iNeuron for endogenous VPS35 and VPS29. Scale bars, 5 μm in (a) and (b). [file 13041_2020_675_MOESM4_ESM.pdf]

**a**

iNeuron; early endosome (Rab5a)

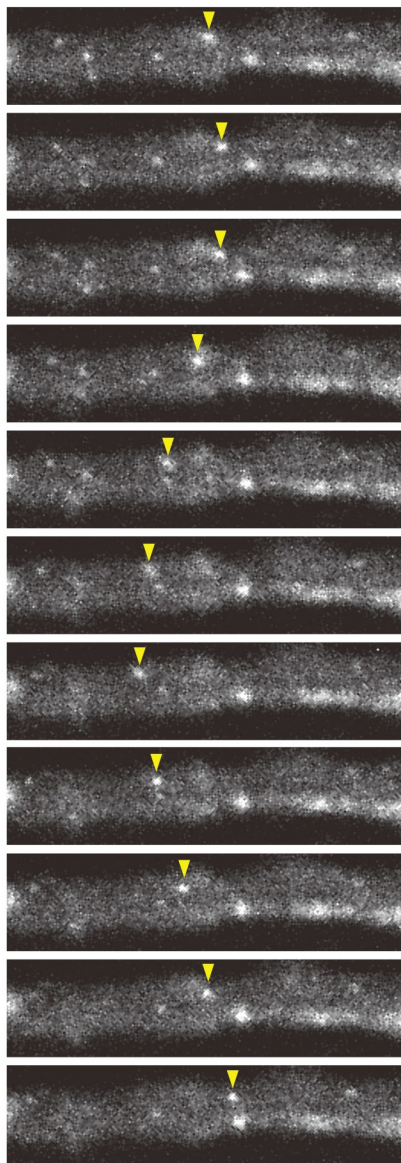**b**

iNeuron; late endosome (Rab7a)

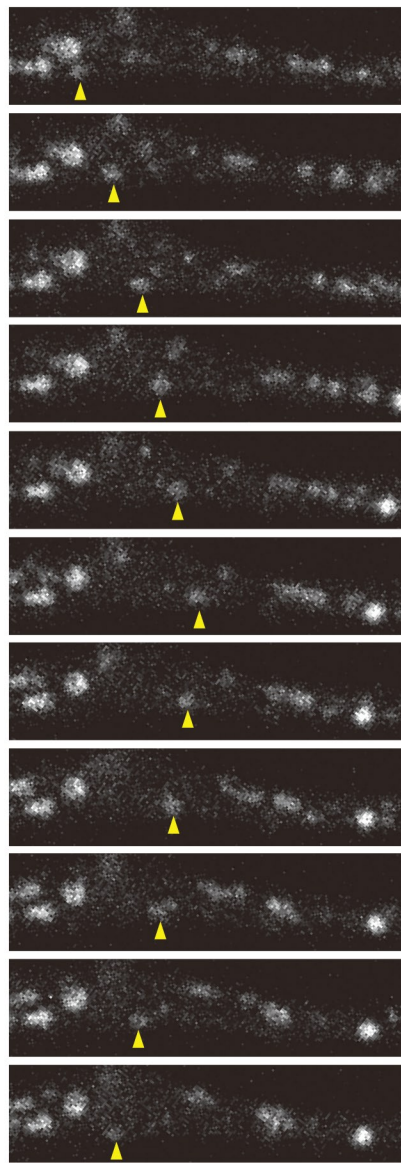

Supplement: Supplementary file 11 — Additional file 11: Movie S7. iPSC-derived neurons (iNeurons) from healthy controls stably expressing RFP-Rab7a were imaged by time-lapse fluorescence microscopy. [file 13041_2020_675_MOESM11_ESM.pdf]

**a**

RFP-Rab5a

fission

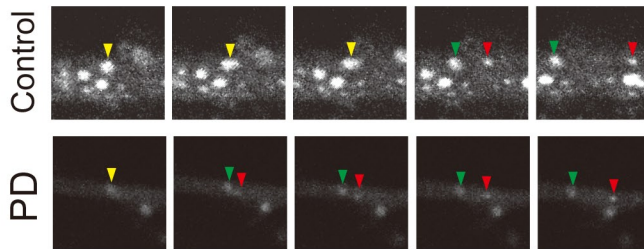

fusion

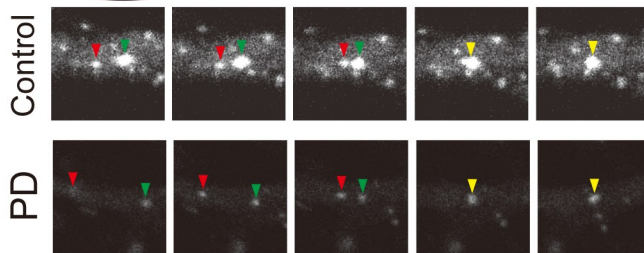

**b**

RFP-Rab7a

fission

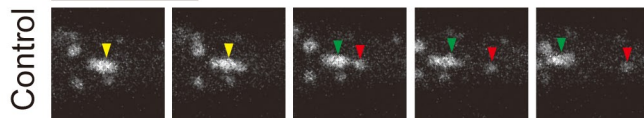

fusion

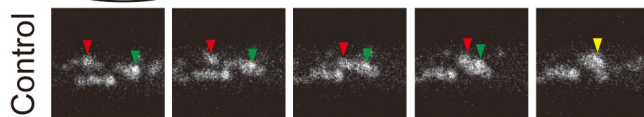

Supplement: Supplementary file 14 — Additional file 14: Figure S5. Endosomal fission and fusion related to the data in Figure 5. (a) RFP-Rab5a in the neurites of control and PD iNeurons was imaged by time-lapse fluorescence microscopy. (b) RFP-Rab7a in the neurites of control iNeurons was imaged by time-lapse fluorescence microscopy. [file 13041_2020_675_MOESM14_ESM.pdf]

**a**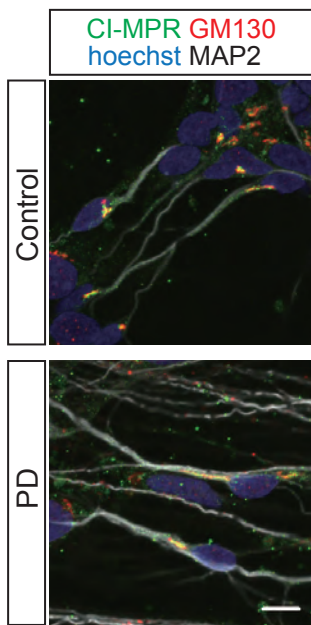**b**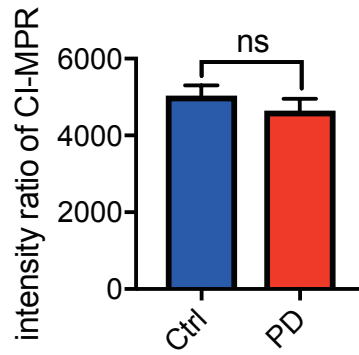**c**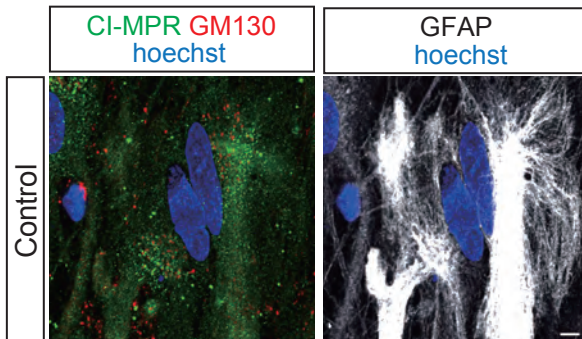

Supplement: Supplementary file 15 — Additional file 15: Figure S6. Localization of CI-MPR in neurons derived from iPSCs (iNeurons) related to the data in Figure 6. (a) Immunostaining of neurons derived from iPSCs (iNeurons) from PD patients and healthy controls for endogenous CI-MPR and the Golgi. Most CI-MPR was localized around the Golgi. (b) Quantification of the results of the localization analysis performed in (a). The figure shows the intensity of CI-MPR staining in neurites (n = 3 per line). There was no difference in intensity between PD and Control groups. (c) Immunostaining of glial cells derived from iPSCs from healthy controls for endogenous CI-MPR, GM130 and GFAP. Data are represented as mean ± SEM; n.s., not significant; Mann–Whitney U-test in (b). Scale bar, 10 μm in (a) 5 μm in (c). [file 13041_2020_675_MOESM15_ESM.pdf]

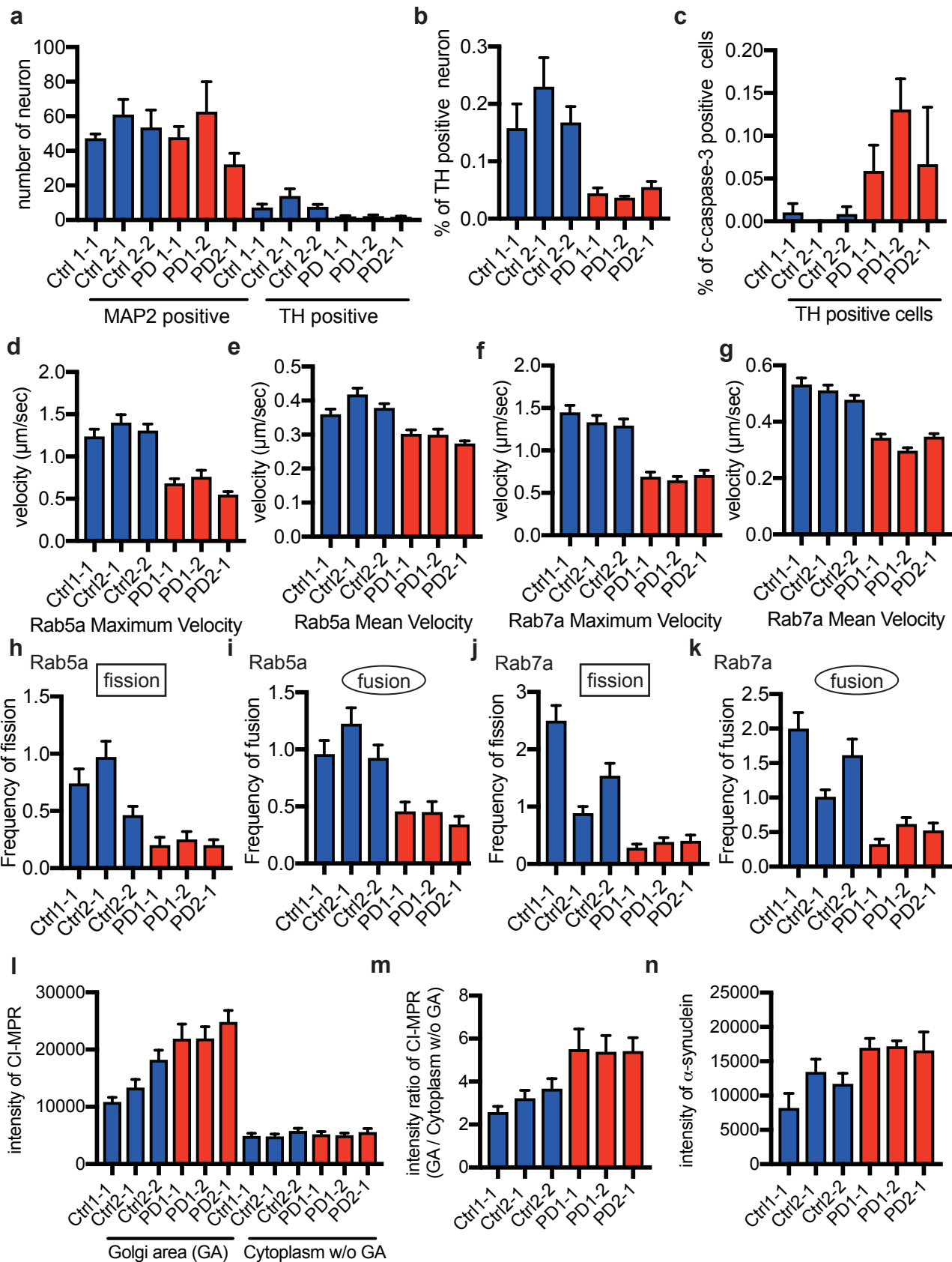

Supplement: Supplementary file 16 — Additional file 16: Figure S7. Experimental results in each iPS line. [file 13041_2020_675_MOESM16_ESM.pdf]
